# Usability and User Experience Testing of a Co-Designed Electronic Patient-Reported Outcomes App (“MyPal for Adults”) for Palliative Cancer Care: Mixed Methods Study

**DOI:** 10.2196/57342

**Published:** 2025-04-24

**Authors:** Panos Bonotis, Pantelis Angelidis, Pantelis Natsiavas

**Affiliations:** 1 Institute of Applied Biosciences, Centre for Research and Technology Hellas, 6th km Charilaou-Thermi Road, Thessaloniki, 57001, Greece, 30 2310257606; 2 Department of Electrical & Computer Engineering, University of Western Macedonia, Kozani, Greece

**Keywords:** mHealth, app, digital intervention, think-aloud test, palliative care, cancer care, patient-reported outcomes, usability evaluation, mobile health, mobile phone

## Abstract

**Background:**

Mobile health technologies have the potential to enhance the management, communication, and overall quality of life of patients with cancer. The MyPal project, using a participatory design approach, aims to provide palliative care support for patients with cancer through an electronic patient-reported outcomes eHealth platform.

**Objective:**

This study aims to evaluate the usability and user experience of “MyPal for adults,” a co-designed palliative care mobile app designed to support adults with cancer.

**Methods:**

Representative users participated in a 4-step usability study using a “think-aloud” protocol, complemented with feature satisfaction, difficulty perceived, and design impression surveys along with a short, structured interview. Participants were also asked to provide quantitative feedback via the postuse System Usability Scale, User Experience Questionnaire, and Post-Study System Usability Questionnaire. The data were analyzed along the lines of the International Organization for Standardization (ISO) 9241‐210 framework.

**Results:**

All participants found the intervention content useful, and they reported satisfactory usability, with a mean Post-Study System Usability Questionnaire score of 2.458 (SD 1.08) and a System Usability Scale score of 68.9. All aspects of the User Experience Questionnaire (attractiveness, perspicuity, efficiency, dependability, stimulation, and novelty) surpassed usability quality benchmarks. The qualitative analysis identified 43 usability issues, primarily related to effectiveness and efficiency as defined in ISO 9241‐210.

**Conclusions:**

In this study, we conducted a usability evaluation of the “MyPal for adults” app, a digital tool designed to enhance the palliative care experience. This approach identifies real-world usability issues, enabling iterative improvements in the eHealth platform’s design. Our findings reveal a user-friendly interface and positive patient experiences. This study emphasizes the need to enhance mobile health platform usability, offering insights to improve digital palliative care.

## Introduction

### Background

Hematological malignancies (HMs) account for about 6.5% of global cancer cases, primarily affecting older individuals around 70 years old [1]. Patients with HMs often experience distressing symptoms, which can lead to a lower quality of life (QoL) [2]. Consequently, palliative care has emerged as a vital health care practice to enhance the QoL of individuals facing serious, life-limiting illnesses, regardless of age or disease stage [3].

Effective palliative care relies on active patient-health care team communication, where electronic patient-reported outcome (ePRO) systems offer substantial promise [4,5]. These systems enable patients to report their health status, including personal evaluations, symptoms, psycho-emotional state, and adverse drug reactions via defined questionnaires. However, despite their potential to enhance patient care, the widespread integration of ePRO systems in routine health care and research remains limited [6].

Although smart device adoption in health care began increasing even before COVID-19 [7], enabling more direct dissemination of health information, complex interfaces often lead to app abandonment, especially among older users. The Google and Apple app stores offer around one million health-related apps, with approximately 300,000 in the mobile health (mHealth) category [8]. Despite this, mHealth app downloads are declining [9,10], raising concerns about their usability and acceptance, influenced by factors like privacy and usability [11].

To address these challenges, co-design approaches have gained interest [12]. Co-design involves collaborative efforts among researchers, designers, and end users who actively contribute to knowledge development, idea generation, and concept refinement [13]. This user-centered approach fosters a deeper understanding of user perspectives [14] and can optimize the development and usability of palliative care ePRO apps.

### MyPal Project

MyPal [15], a collaborative H2020 research project funded by the European Commission, uses eHealth technologies to support adults and children with cancer through advanced patient-reported outcome systems. Its primary aim is to develop and clinically assess 2 new ePRO-based palliative care interventions for adults and children, along with their carers, and health care professionals (HCPs) to enhance their QoL [16], improve effective communication, and management of the disease. MyPal uses a user-centered, iterative methodology involving end users from the project’s inception to design various platform components. The design of the MyPal platform and its features were aligned with the needs of patients receiving palliative care for hematologic malignancies, as identified through research conducted during the early stages of the MyPal project. These include symptom management tools (eg, pain and fatigue tracking and spontaneous symptom reporting), psychosocial support features such as customizable motivational messages and emotional well-being assessments via ePROs, and tools for personalized care planning allowing patients to set individual goals and preferences. In addition, the app facilitates advanced care planning discussions by providing educational resources that address both treatment and QoL considerations.

The MyPal platform for adult patients consists of a mobile app for the patient, a web app for the HCP, and tools for the administrator. MyPal also supports young patients (children and adolescents) and their careers in the CHILD study [17] with a serious game for patients, a mobile app for the carer, and a web app for HCPs. These interventions and their associated technologies are to be tested in a randomized clinical trial conducted across 5 countries (Greece, Germany, Italy, Sweden, and the Czech Republic).

**Figure 1.**
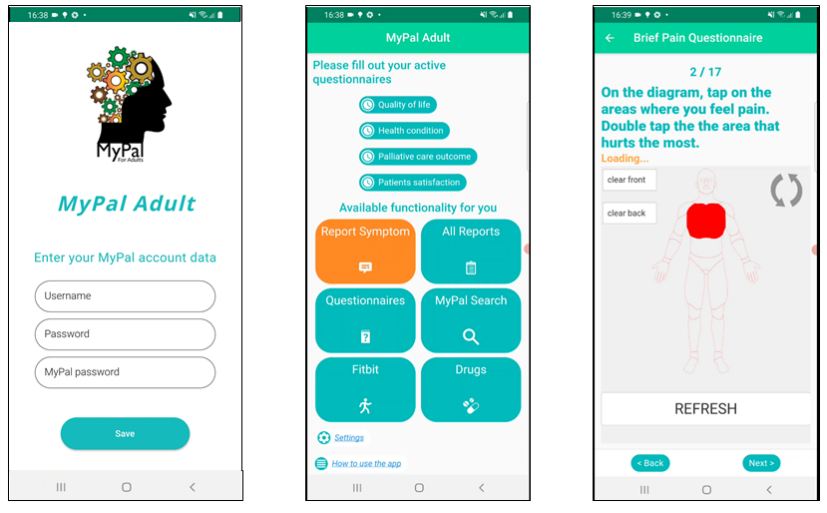
The MyPal mobile app for adult patients: login page, home screen, and example question from the electronic patient-reported outcome module. The interface tested was in Greek; the version shown here is in English to facilitate clear communication of the app’s design and functionality.

This usability study focuses on the “MyPal for adults” app [18] which has an end goal to facilitate (1) better self-management of the HM disease symptoms, and (2) timely assessment of the reported symptoms or QoL deteriorations by treating HCPs. Technical components are demonstrated in an openly available video [19]. The MyPal adult platform includes a mobile app (Figure 1) that supports periodic and spontaneous reporting of physical and psycho-emotional symptoms, using standardized ePROs for periodic reporting and custom electronic forms for spontaneous reporting. These reports, along with the standardized ePRO data, are directly accessible to HCPs via their dedicated platform. HCPs can view reports, track the status of questionnaires for each patient, and be alerted to any symptoms that meet predefined thresholds, ensuring timely clinical intervention. The app also offers features such as medication management, where the patient can set reminders for dosage intake, an HCP-tailored disease, and health information search engine, personalized motivational messages that are sent to patients based on their needs collected via screener questionnaires at specific time points, and tracking of lifestyle parameters like physical activity and sleep quality. Additionally, “MyPal for adults” provides secondary supportive features that are optional for use, including an app tutorial, tech support chat, and contact information for local MyPal liaisons. Overall, the app is provided in 5 languages: Greek, Italian, Swedish, Czech, and English (only for demonstration).

### Objective

This study aims to evaluate the usability and user experience (UX) of a co-designed, midfidelity version of the “MyPal for adults” mobile app using a 4-step framework, focusing on assessing content, functionality, and barriers experienced by end users.

In that context, the research questions addressed are: Is “MyPal for adults” usable, comprehensible, satisfactory, and acceptable to end users? If not, what are the usability and satisfaction issues in terms of number, type, and severity as evaluated by patients?

## Methods

### Explorative Usability and UX Assessment

**Figure 2.**
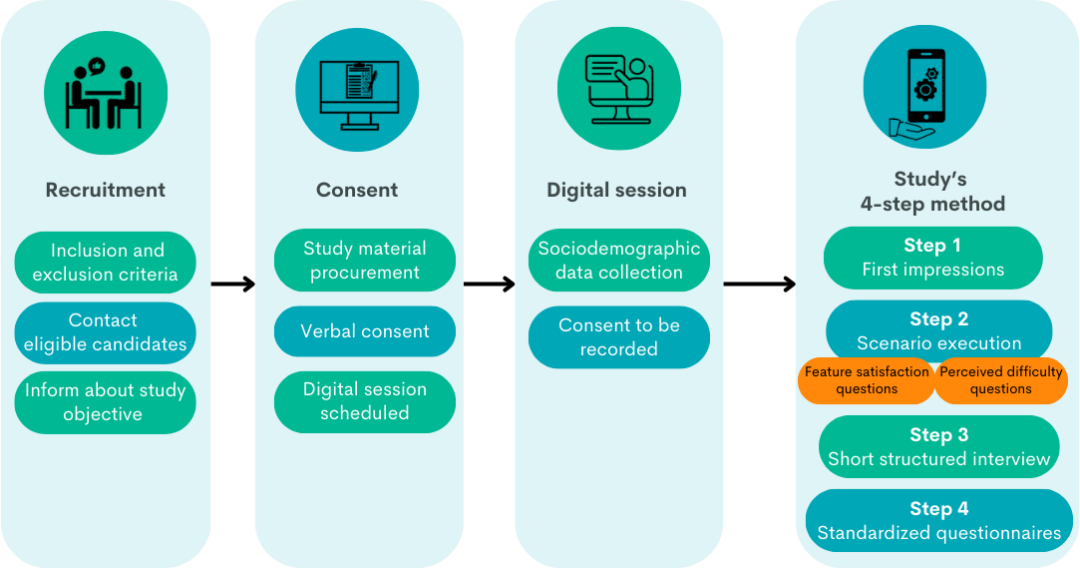
Participant journey in the usability study.

We chose an exploratory approach to assess usability and UX through a 4-step process, including a coaching “think-aloud” protocol [20], structured interviews, and follow-up usability and UX measuring questionnaires: the Post-Study Systems Usability Questionnaire (PSSUQ) [21], the System Usability Scale (SUS) [22] and the User Experience Questionnaire (UEQ) [23] known for their established effectiveness in the literature. This process simulated a patient’s journey in the MyPal Adult intervention study, as described in its protocol [2] (Figure 2). Given the COVID-19 pandemic restrictions and the Greek National Health System’s advisories against hospital visits, we conducted all assessments remotely using digital tools.

Usability, as per the ISO (International Organization for Standardization) usability framework (ISO 9241‐210), is “the extent to which a product can be used by designated users to achieve specific goals with efficiency, effectiveness, and satisfaction, in a specific context use” [24]. The criteria of this framework are effectiveness, efficiency, satisfaction, and context of use. Typically, the concept of usability also covers comprehensibility, learnability, functionality (operability), attractiveness, appropriateness recognizability, accessibility, user error protection, and interface aesthetics [25].

As described in ISO 9241‐210, effectiveness is the accuracy and completeness with which users achieve specified goals, such as symptom reporting. Efficiency refers to the resources used in relation to the results achieved, like the time needed to report a symptom. Satisfaction is defined as the “extent to which the user’s physical, cognitive, and emotional responses that result from the use of a system, product, or service meet the user’s needs and expectations” [24].

UX is defined as “user’s perceptions and responses that result from the use or anticipated use of a system, product, or service.” [26]. Good UX not only contributes to higher work motivation and performance but can also affect the well-being of users [27]. Achieving acceptable UX or usability requires systematic evaluation and iterative design adjustments as the app matures [28].

### Participant Recruitment

Participants for this study were recruited through nonprobability methods, a mix of purposive sampling to target relevant participants, and convenience sampling to address recruitment challenges during the COVID-19 pandemic. They were recruited based on predefined inclusion criteria in the project’s randomized clinical trial protocol [2]. Indicatively, adults (18 years or older) diagnosed with hematologic cancer, who were scheduled to receive or had previously been exposed to any line of treatment, and were users of an internet-connected device (smartphone or tablet). Exclusion criteria included patients requiring immediate referral for specialized palliative care or with a life expectancy of less than 3 months. To this end, given our institution’s close collaboration with ELLOK (Greek Federation of Cancer Patients), members were contacted via telephone and email, facilitated by MyPal’s clinical liaisons, ensuring the participation of members who are patients with hematologic cancer. Finally, since ELLOK had previously procured volunteers to consult on and test earlier prototypes, we added an exclusion criterion stating that no participant should have had any prior experience interfacing with the midfidelity app in focus. This exclusion criterion was added to ensure that all participants would be “naïve” users, that is, users who have no training or experience with the app, as would be the case for a patient enrolling in the MyPal study.

To address the recruitment difficulties posed by the pandemic and in consultation with our clinical liaisons, the inclusion criteria were expanded to include individuals with other chronic diseases sharing care needs similar to hematologic cancer (eg, autoimmune skin conditions and chronic pulmonary diseases) and recent hospital visits in the last 6 months were also planned to be included as a countermeasure.

### Data Collection

#### Global Process

In alignment with the MyPal Adult intervention study protocol, interested and eligible candidates were informed about this usability study objective and the MyPal project goal, its technologies, and their functionalities, and provided with a detailed informational sheet and contact details for any inquiries. The consent process was also conducted remotely using digital tools due to the COVID-19 pandemic restrictions. After confirming their participation in this usability study, they were emailed with every material a patient joining the MyPal Adult intervention study would receive, specifically the link to download the app, a user manual, and a quick-start guide, offering a summary of the project’s goal and the platform capabilities. They were also asked to confirm the appointment for the usability session. Verbal consent was obtained once more by the moderator of the usability study prior to the session initiation.

**Figure 3.**
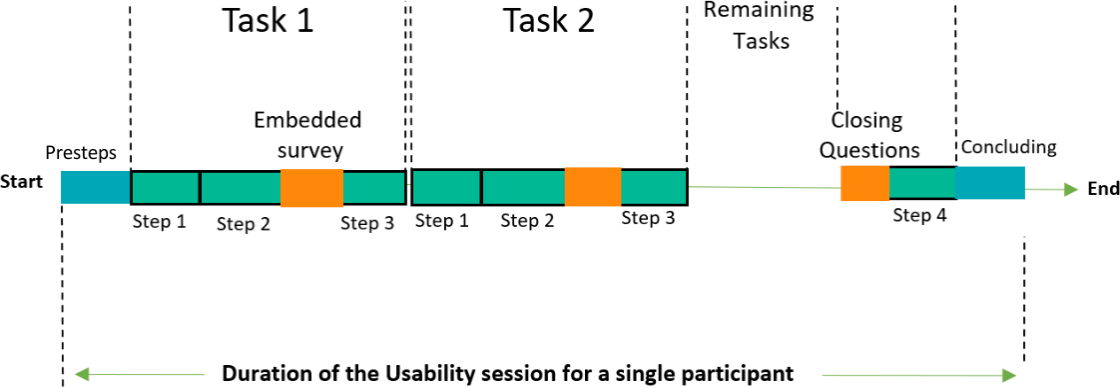
Step sequence in a single think-aloud session.

Each participant completed a single 55- to 70-minute teleconference session, following a structured task-based use scenario (Figure 3). The moderator used a PC with a webcam and the Zoom (Zoom Communications, Inc) platform to record facial expressions and facilitate the sharing of the mobile device screen. Participants were asked to install the Zoom app on their smartphones and laptops prior to the session to enable screen sharing from their smartphones and record facial and vocal interactions via their laptops. For the app testing, participants used their own Android devices; however, the research team had a backup Android device available if needed.

#### Preparatory Steps

At the beginning of each session, participants provided sociodemographic information, including age and sex. They also answered 2 multiple-choice questions about their smartphone skills, based on their comfort level and experience with using smartphone apps, including frequency of use, ability to navigate apps, and familiarity with mobile technology in general, and 3 questions on smartphone app use (Multimedia Appendix 1). Following the introduction, the usability study’s context, task completion logic (described in the task “scenario” in Multimedia Appendix 2), and the “think-aloud” process were explained in detail. An example task was also presented to familiarize participants with the “think-aloud” method.

#### Step 1: First Impressions

During the task “scenario” execution, participants navigated to various functionalities’ screens. The moderator inquired about the screens’ general eye-catching attributes and the users’ initial impressions. For each screen, 2 questions were posed: (question 1.1) “What is your initial impression of this screen?” and (question 1.2) “How do you rate the design and information organization?” To gain more detailed insights, a third question was added for each screen (question 1.3): “What actions do you think you can perform on this screen?”

#### Step 2: Task “Scenario” With an Embedded Survey for Satisfaction and Perceived Difficulty

The second step involved executing tasks within the “scenario,” where participants were encouraged to think aloud to identify usability issues, aligning with the “think-aloud” protocol. The “scenario” included in total of 16 tasks (Multimedia Appendix 2). Some of the tasks included multiple-choice questions in the 5-Likert and 7-Likert scale [29], to gather quantitative data on perceived difficulty and satisfaction with specific functionalities. While the satisfaction questions were inquired for every feature in the app, (mainly focusing on modules and elements regarded as important early on in the design ideations), the perceived difficulty questions were more targeted. Difficulty-perceived questions were only inquired during tasks that had performed poorly based on the results of prior assessment procedures (validation, rehearsal testing) within the MyPal project. In accordance with the “think-aloud” protocol, moderators worked with participants during task execution, meaning they would intervene to prompt participants to verbalize their thoughts, or “think-aloud.” When a participant struggled with a task, the moderator noted it down and provided guidance. If participants repeatedly encountered issues with a specific action leading to task failure, moderators inquired about the problem’s nature to capture participant perceptions and experiences.

Participants’ verbalizations about their preferences or dislikes regarding the PROs content were not considered, as it was not the study’s purpose. Nonetheless, participants were encouraged to voice their thoughts while going through the content to uncover potential user interface (UI) inconsistencies.

#### Step 3: Short Structured Interview

After completing the final task in the “scenario,” participants were given the opportunity to add anything to their responses regarding feature satisfaction from step 2 and to address any follow-up questions related to the usability evaluation and overall UX. Additionally, 3 extra questions were posed as part of the structured interview following the last task of the “scenario:” (question 3.1) “How do you perceive the secondary complementary supportive features? Would you use them?” (question 3.2) “What would be your first action with this app?” and (question 3.3) “Do you feel the app expects something from you?”

#### Step 4: Standardized Questionnaires

Before concluding each session, quantitative feedback was gathered from participants using a set of well-established and validated questionnaires. These included the SUS, a 10-item questionnaire rated on a 5-point Likert scale ranging from Strongly Disagree to Strongly Agree, which assesses the overall usability of a system [22]. The UEQ measures 6 distinct dimensions of UX—attractiveness, perspicuity, efficiency, dependability, stimulation, and novelty—using a 7-point semantic differential scale [23]. Finally, the PSSUQ, which is rated on a 7-point scale from Strongly Disagree to Strongly Agree, evaluates user satisfaction with system usability, covering areas such as task ease, efficiency, and interface quality [21]. Participants were then given the option to submit either only the PSSUQ or all 3 questionnaires.

### Data Analysis

Steps 1 and 3 (“first impression” and “short, structured interviews”) underwent inductive thematic analysis [30], involving transcription and translation of all participant responses into English, along with the calculation of standardized questionnaire scores. In step 2 (task “scenarios”), a deductive analysis aligned with the ISO framework categories was performed [30].

### Ethical Considerations

The study received ethical approval from the Bioethical Committee of the Center for Research and Technology Hellas (ETH.COM-61). The study involved human participants and adhered to institutional and ethical guidelines for research involving human participants. Informed consent was obtained from all participants after they were provided with clear information regarding the purpose of the study, procedures involved, data handling, and their right to withdraw at any time without penalty. No waiver of consent was applied. Privacy and confidentiality were maintained throughout the study. All data collected were fully anonymized before analysis, and no personally identifiable information was stored or used in reporting. Any potential identifiers in screenshots or visual material were removed or blurred. Participants did not receive any form of remuneration or compensation for their participation in the study.

## Results

### Participant Demographics

A total of 9 participants took part in the usability and UX testing. They were recruited from 3 regions in Greece—Thessaloniki, Karditsa, and Heraklion—for feasibility and proximity reasons. The study group included 4 individuals with chronic lymphocytic leukemia (CLL), 2 senior citizens, and 3 adult representative users afflicted by a range of chronic ailments. The gender distribution was balanced, with 4 (44%) women and 5 (56%) men. Participants’ ages ranged from 27 to 68 years old, with a mean age of 49.1 (SD 13.6) years, and a median of 17 (IQR 42-59) years. Regarding smartphone skills, participants self-reported varying levels of proficiency: 1 (11%) participant reported poor skills, 2 (22%) participants reported low skills, 2 (22%) participants had medium skills, 1 (11%) participant had high skills, and 3 (33%) participants considered their skills to be perfect. In terms of prior experience with similar apps, 2 (22%) participants had used health-related apps before, while the majority of participants (7/9, 78%) had no previous experience (Table 1).

**Table 1.** Participants’ sociodemographic data.

Characteristics	Value (N=9)	
Gender, n (%)		
Female	4 (44)	
Male	5 (56)	
Age (years), mean (SD)	49.1 (13.6)	
Smartphone skills, n (%)		
Poor skills	1 (11)	
Low skills	2 (22)	
Medium skills	2 (22)	
High skills	1 (11)	
Perfect skills	3 (33)	
Prior experience with similar apps, n (%)
Yes	2 (22)	
No	7 (78)	
Participant’s diagnosis, n (%)
CLL^a^	4 (44)	
Chronic arthritis	1 (11)	
COPD^b^	3 (33)	
HS^c^ stage 3	1 (11)	

a CLL: chronic lymphocytic leukemia.

b COPD: chronic obstructive pulmonary disease.

c HS: hidradenitis suppurativa.

### Participants’ Feedback

#### Step 1: First Impressions

##### Overview

This section presents the results from open-ended questions gathered during the first impressions collection step, translated into English. Each participant is referenced by an assigned ID (eg, P1 and P2). The analysis of these initial impressions is based on themes that emerged during the inductive thematic analysis.

##### Question 1.1: “What Is Your Initial Impression of This Screen?”

Every functionality and screen received a positive first impression. No one expressed any major dislikes or was annoyed by something particular in the design. The only negative comment was about the app’s icon:

*I don’t particularly like that the icon’s portraying a human head that is black like it is burned. Also, the gears appearing like they are inside the head makes me cringe a little bit.*

[P4]

##### Question 1.2: “How Do You Rate the Design and Information Organization?”

As question 1.2 significantly overlapped with question 1.1, participants had already developed an impression of each screen’s design by the time question 1.1 was posed. All participants neutrally accepted every screen in each functionality, with minor feedback primarily related to button placement and text entry field colors.

When analyzing participant responses, 2 distinct user groups emerged: young users (25-35 years of age) and senior users (45-70 years of age). While younger participants desired a more modern app design, they found the app’s current design very user-friendly.

*It would be very good if there was an additional UI with a more modern design, like with a side menu etc but I guess this would be more helpful with older patients as well. I don’t really mind it as long as it proves easy-to-use and useful. Which I guess it will ... at least it seems that way.*

[P8]

The color palette of the app and the simplicity of the design were very much appreciated by the more senior users.

*I really like the colors, they are quite soothing, and give me the “health care” vibe. Everything seems quite simple and straightforward.*

[P2]

##### Question 1.3: “What Actions Do You Think You Can Perform on This Screen?”

The main purpose of this question was to determine if the context of use for each functionality was evident from the screen’s appearance and whether the action-probing screens were distinguishable from the informing and data-viewing screens. Almost all participants correctly identified the main purpose of each screen, except for one participant (P9), who was a senior patient with limited technology experience.

*I understand that the purpose of this screen is to help me manage my disease. I guess it will ask me somehow about my medication. Can I speak to it? I don’t understand how to do this or what to do.*

[P9]

### Step 2: Task “Scenario” With an Embedded Survey on Satisfaction and Perceived Difficulty

We identified 43 problems in the task “scenarios,” which were classified according to the categories defined as part of the ISO 9241‐210 framework. The task can be found in Multimedia Appendix 2. Most problems (10/43, 23%) occurred during questionnaire submission at task 3. Other tasks with notable problems were drug entry (5/43, 12%) and reminder setting in the drug module (8/43, 19%) at tasks 11 and 12, respectively.

Table 2 presents the problems mapped to the main categories defined by the ISO usability framework, an example for each, and the transcript or description of the observation. It also presents the task where the problems occurred and the type of the problem. Some tasks could not be completed because of programming errors or bugs. When problems were caused by programming errors, they were classified as N/A (not applicable) in each ISO framework category and the task was considered failed. In those cases, a screenshot was displayed of the broken feature to enable the continuation of the study.

About 30% (13/43) were classified as “effectiveness” and took place mainly at the “add a drug” task, where users were asked to log their medication. A total of 23% (10/43) were classified as “efficiency” and occurred mainly at the registration process during the baseline filling-out questionnaire mainly affected by the repetition of text at each step of the registration process. In the MyPal Adult intervention study, prior to the “MyPal for adults” app’s regular use, a baseline data collection is taking place immediately after logging into the app for the first time, as part of the app’s registration process. The registration process was a relatively new feature, that was not thoroughly tested prior to this study, and problems were expected. It is presented in a step-by-step manner guiding the user to complete all baseline questionnaires and configure app initial settings. In addition, a notable efficiency issue was related to task 16, and the time needed to locate the emergency contact number of the local liaison team in the app. A smaller portion (9/43, 21%) related to “satisfaction” and was mainly noticed in the medication reminder setting task, which was perceived as overly complex by most participants, hindering their satisfaction with it. Two problems were classified as context of use. These problems were identified by a single participant, and both could be explained by the fact that this participant was a senior user, not experienced with mobile apps. The problems coded as the context of use included the following: (1) the participant tried to use the MyPal patient-tailored disease-related information search engine to “ask a question” (expecting a chat function) to the clinicians and (2) the size of some UI elements, that is, the module buttons, text displayed. Finally, 21% (9/43) of the problems were classified as N/A and were related to technical issues, such as software bugs or errors, that were outside the scope of usability-related classifications (effectiveness, efficiency, etc). These technical issues arose from system updates occurring during the usability testing and could not be classified according to the ISO usability framework. Most of these problems occurred mainly in the Fitbit module when attempting to view the sleep data, setting drug reminders, and accessing the patient-tailored disease-related information search engine.

**Figure 4.**
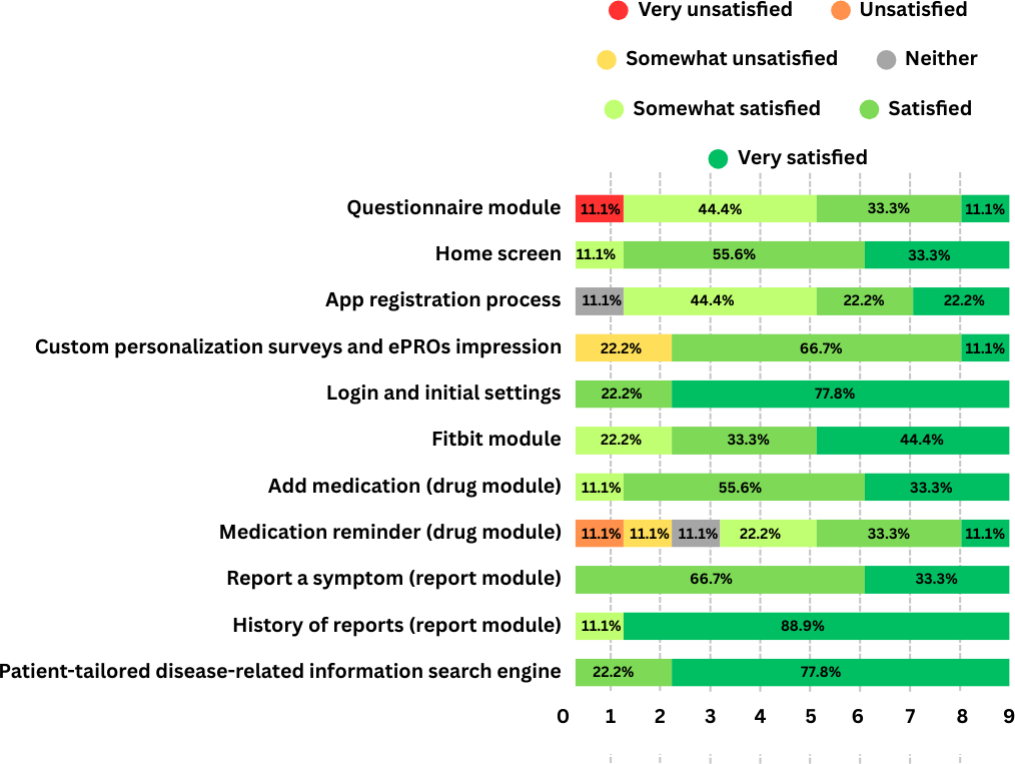
Feature satisfaction. ePRO: electronic patient-reported outcome.

In Figures4  5, we present the results of the feature satisfaction and perceived difficulty questionnaires which complemented the usability task “scenario.” The question formulation can be also found in Multimedia Appendix 2.

As shown in the figures, most of the functionalities of the app were rated as satisfactory and easy to use. However, the medication reminder feature stood out as overly complex for some participants, leaving a portion either unsatisfied or indifferent. Another noteworthy observation concerns the questionnaire module. While most participants found it generally satisfactory, they struggled to locate it quickly, suggesting the need for a more intuitive or descriptive heading. Regarding the ePROs and questionnaires overall (including personalization surveys and symptom reporting), some dissatisfaction and difficulty were noted, largely due to efficiency issues. These stemmed from repetitive text at each question and high question volume, which impacted the UX, and even had some participants thinking it was a technical bug with the system.

**Table 2.** Problems with the app, classified according to the International Organization for Standardization framework.

ISO^a^ categories	Problems (n=43), n (%)	“Scenario” section and task number (example)	Verbalization or observation (example)	Problem detected (example)
Effectiveness	13 (30)	App registration process (task 2)	“So, I can see here the clinics communication information, so I guess I tap on next”	Setting up the notification timing choice was not visually clear and as a result the participant did not notice it and moved on.
Efficiency	10 (23)	Custom personalization surveys and ePROs^b^ impression (task 3)	“Why are the same texts repeated? Wouldn’t it be better to have them all grouped so the texts wouldn’t have to be repeated?”	There are a lot of seemingly repeated questions in a questionnaire, where only a small phrase would change, and all the other wordings would stay the same.
Satisfaction	9 (21)	Login and initial settings (task 1)	“Is this normal, or is it a bug? In any case it should not be like that”	Password needs to be protected. When the passwords are entered, they could be hiding with asterisks.
Context of use	2 (5)	Patient tailored disease-related information search engine (task 14)	Participant is entering a question directed to his clinicians	The participant did not understand that this is a search engine.
N/A^c^	9 (21)	Fitbit Module (task 9)	“It doesn’t work. I am pressing the button but I cannot see my sleep data”	There is a technical issue with a specific button, the participant cannot watch his sleep data.

a ISO: International Organization for Standardization.

b ePRO: electronic patient-reported outcome.

c N/A: not applicable.

**Figure 5.**
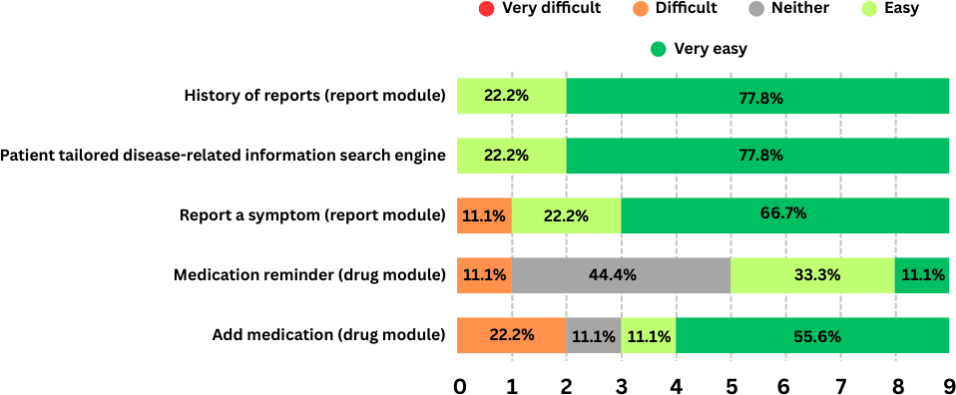
Task difficulty.

### Step 3: Short Structured Interview

This section presents the results from the open-ended questions, translated into English, collected during the structured interview process that followed the task-based “scenario.”

#### Question 3.1: “How Do You Perceive the Secondary Complementary Supportive Features? Would You Use Them?”

All participants expressed their willingness to use every complementary supportive feature. The tech support chat was very appreciated, as well as the app’s manual.

*I really like the fact that the manual of the app is in the home screen and so easily accessed.*

[P3]

*I really appreciate the chatting service for technical support. I feel like I could do this.*

[P7]

#### Question 3.2: “What Would Be Your First Action With This App?”

This question aimed to determine if participants would prioritize pending questionnaires. However, participants’ responses were inconclusive regarding this objective because all users, being new to the app, expressed a desire to explore and become familiar with it instead of immediately focusing on the active pending questionnaires.

*I would like to familiarize myself with the app at first. I would just browse through everything and start playing with it, I guess.*

[P8]

*I would most likely first enter my medications and set reminders for them. Then I would probably report something retrospectively.*

[P2]

Only 2 users expressed the desire to first navigate to the questionnaire module or the questionnaire pending list so they can verify they have completed everything is expected from them:

*I guess first step is to familiarize myself with the app and make sure that I have reported everything I must since I can see in the pending list that there are a few questionnaires available for me.*

[P4]

*Well, I am a gadget geek so I would want to explore the whole app. But first I would navigate to the questionnaire module to make sure everything is set.*

[P1]

#### Question 3.3: “Do You Feel the App Expects Something From You?”

This was a follow-up to question 2, ensuring that pending questionnaires were clear to all users. All participants except one did not grasp the main purpose of the pending questionnaire list on the home screen. The participant who understood its purpose admitted it was not obvious and required guidance from the moderator.

*Yes, of course it is expecting me to answer these pending questionnaires displayed in this list. But I have to say if you had not asked me before I would not notice it right away. I would eventually but not right away.*

[P5]

### Step 4: Standardized Questionnaires

Following every session, the participants were asked to submit a follow-up questionnaire that included the postuse SUS, the UEQ, and the PSSUQ. All participants submitted every questionnaire. The mean scores of each questionnaire are presented in Table 3.

**Table 3.** Poststudy standardized questionnaire scores.

Standardized questionnaires	Value
PSSUQ^a^**^,^**^b^	
SYSUSE^c^ score, mean (SD)	2.14 (0.74)
INFOQUAL^d^ score, mean (SD)	2.70 (1.09)
INTERQUAL^e^ score, mean (SD)	2.77 (1.48)
Overall PSSUQ score, mean (SD)	2.46 (1.08)
SUS^f^^,g^ score	68.9
UEQ^h^^,i^ means, mean (SD)	
Attractiveness	1.667 (0.574)
Perspicuity	1.444 (0.728)
Efficiency	1.500 (0.671)
Dependability	1.361 (0.6)
Stimulation	1.528 (0.52)
Novelty	1.306 (0.825)

a PSSUQ: Post-Study System Usability Questionnaire.

b Scale: 1=strongly agree or best score to 7=strongly disagree or worst score.

c SYSUSE: System Usefulness.

d INFOQUAL: Information Quality.

e INTERQUAL: Interface Quality.

f SUS: System Usability Scale.

g Scale: 0=worst usability to 100=best usability.

h UEQ: User Experience Questionnaire.

i Scale: –3=worst to +3=best.

## Discussion

### Principal Results and Findings

This study assesses the usability and UX of the “MyPal for adults” app in the early phases of the MyPal project. Results from the usability test yielded a marginally positive SUS score of 68.9, as per Brooke [22]. UX results were favorable, as evidenced by UEQ means and user feedback. PSSUQ also showed a positive outcome (mean 2.46, SD 1.08), with higher ratings for usefulness compared to information quality and UI. While all quantitative data indicated an acceptable score, a closer examination of participant responses revealed the primary issues with the app. User feedback emphasized the importance of tailoring specific interface elements, such as wording changes (eg, “Questionnaires” to “My Questionnaires”). Additionally, technical bugs in 2 features and repetitive text in ePRO screens reduced satisfaction and utility. Furthermore, certain functions, particularly the Medication module, were seen as overly complex, requiring refinement to enhance ease of use. Despite these challenges, users expressed a desire to engage with the app and appreciated its potential once they became familiar with its features.

Findings from step 1 confirm that all participants found the presented information comprehensible, with a clear organization of information. However, participants made numerous proposals, suggesting a potential need for a more personalized interface that accounts for age-related preferences. Older users appreciated the app’s simplicity, while younger users preferred a more modern and visually engaging design. The feedback primarily focused on button placement, header wording, and contextual text. Given that these differences between age groups were consistently observed throughout the study, a dual-tiered UI could provide an effective solution. Such an approach would allow users to choose between a more visually appealing, modern interface and a minimalist, straightforward design better suited for older adults or those less familiar with technology. This tailored UI could help mitigate issues related to horizontal comprehension and ensure that every module’s screen has its purpose understood by all participants.

Findings from step 2 revealed more major issues in the drug reminders and emergency contact detail modules compared to the minor issues found with the app’s core functionality, the ePRO submission, which mainly related to wording and information displayed refinements. Participants found the medication reminder function confusing and overly complex and indicated they were unlikely to use it. Interestingly enough, during the “think-aloud” part of the study, most participants also dismissed this as an issue by pointing out that they already have a system and keep a medication log with reminders in place, making this feature possibly redundant. Locating emergency contact information was challenging for all participants, which in an emergency situation, could prove vital. Participants suggested a more obvious approach, where the information can be found at the top of the home screen, which arguably could hinder usability by limiting the available screen to essential app functionalities and should be further explored. Additionally, 2 modules faced technical problems: (1) the search engine module and (2) the activity data display in the Fitbit module. The search engine module experienced unexpected bugs because the study was conducted on the development server, while the Fitbit module had difficulties connecting to the Fitbit server during the project’s early development phase.

In general, based on the questionnaire results, the participant’s attitude, and our overall impression and experience of the usability study, participants enjoyed using the app, and in many cases, they complemented their feedback with suggestions for UI-related details and expressed concerns about module titles or text. For instance, “Questionnaires” could be improved to “My Questionnaires” to clarify its purpose. An interesting realization was the importance of translating paper-based PROs into digital “ePROs” successfully. Unlike paper-based PROs presented on a single sheet, ePROs in the “MyPal for adults” app had individual screens for each question, leading to perceived information redundancy and concerns about falsely perceived technical issues. Participants also raised concerns about HCPs’ ability to respond promptly to MyPal reports due to their busy schedules, which could affect the UX of patients, aligning with previous research on chronic pain monitoring [31].

Findings from step 3 indicate that participants were eager to explore and familiarize themselves with the app before engaging with specific tasks, such as completing pending questionnaires, an encouraging sign of positive anticipation for using the system. Additionally, many expressed a desire to explore the app, set up medication reminders, and retrospectively report symptoms. The accessibility of the app’s manual was highly appreciated, especially by more senior participants, who also were positively surprised, and even relieved, when they realized the app had its own tech support functionality. Further investigation revealed that senior users anticipated complex background computations, and the availability of tech support reassured them. One of the app’s core functionalities, facilitating ePRO submissions, was supported by in-app reminders and notifications, as well as a list of pending ePROs prominently displayed on the home screen to draw users’ attention upon opening the app. While some participants quickly identified the pending questionnaire list, others required additional guidance, underscoring the need for clearer design cues or onboarding processes to ensure critical tasks were not missed.

Finally, the importance of iteration in the development cycle must be highlighted since all participants had problems with the presentation of the ePROs, and almost all participants but one missed the pending list of questionnaires, present on the home screen of the app. Although this could in part be attributed to the actual question which was formulated in a way to not betray its purpose, the fact that it was missed completely by almost every participant (all but one) suggests that a redesign of the app following the principles of UX is needed.

### Challenges and Limitations

Study limitations include programming errors that interrupted tasks and potentially led to the loss of usability information. These errors endured due to concurrent bug fixes conducted alongside the study. The scheduling challenges related to usability testing caused by COVID-19, compounded by the Greek National Health System’s discouragement of hospital visits, resulted in significant delays. Consequently, participants faced programming errors that impeded their ability to complete tasks without issue.

Beyond these limitations, the main challenges were related to the study participants. The purposive sampling method used in this study, while suitable for targeting specific user groups, does introduce inherent selection bias. Participants were chosen based on their medical conditions and alignment with clinical trial inclusion criteria. This limits the generalizability of our findings to the broader population but remains relevant to the specific user group we studied. Furthermore, the nature of usability testing and the absence of blinding may have influenced participant feedback, as users were aware they were interacting with the MyPal app. We aimed to mitigate this by simulating real-world use cases via a use scenario, where users would receive a thorough briefing during onboarding, conducted by a recruiter physician to explain the functionalities of the app. We also encouraged participants to provide candid and constructive feedback throughout the process.

Additionally, the participants had a very diverse level of smartphone skills which along with the age variation, posed a limitation. Age is a determining factor in the use of mobile app products, especially considering neurodegenerative conditions that affect older individuals may significantly impact dexterity, and thus navigation. While the study did not focus on participants with such conditions, the age range of 27 to 68 years presents its own challenges. However, the median of 17 (IQR 42-59) years suggests that the majority of participants were within a relatively concentrated age group, minimizing the impact of an extensive age gap.

Finally, participant recruitment was arduous due to the constraints imposed by the COVID-19 pandemic. Initially targeting users with hematologic malignancies and more specifically CLL or myelodysplastic syndrome, recruitment difficulties led to a shift toward enlisting senior patients coping with other chronic diseases that necessitate periodic hospital visits. While the original objective was to secure 10‐15 participants, the study ultimately included 9 participants. Faulkner [32] has demonstrated that groups of 10 users can identify an average of 95% of usability problems, mitigating concerns regarding sample size. It is worth noting that the study’s findings possess limited generalizability but can be partially applied to self-management mHealth systems using ePROs within the context of palliative cancer care, with a focus on a dedicated mobile app tailored for patients with cancer.

### Comparison With Prior Work and Future Directions

Our usability evaluation of the “MyPal for adults” app demonstrates its potential to improve patient-provider communication, supporting the broader scope of palliative care for patients managing chronic illnesses. Comparing our findings with prior research in the field of eHealth apps for palliative care, several key insights emerge. First, this study aligns with previous research that emphasizes the potential of eHealth apps to improve communication between patients and HCPs in palliative care settings [33]. Through the MyPal app, participants in this study reported improved channels for information exchange, empowering them to actively participate in their care decisions. This echoes findings from related studies [33,34], which have also highlighted how eHealth apps can bridge the communication gap in palliative care, providing a convenient and accessible platform for patients to engage with their health care providers.

While our usability evaluation yielded positive outcomes, it is important to note that, similar to much of the prior work in this field [33], this study primarily involved testing, feasibility assessments, and acceptability studies on relatively small patient populations. A comprehensive evaluation that can impact patient outcomes, including symptom management, QoL, and overall satisfaction with care, necessitates the execution of rigorous clinical trials [35]. This phase of assessment represents the final critical stage in establishing the app’s effectiveness and suitability for integration into palliative care practices, as is the plan with the “MyPal for adults” app.

Future research should aim to broaden the use of the MyPal app to include a more diverse range of patients in palliative care. Additionally, the integration of the app with existing health care systems is crucial to enhance its functionality, providing HCPs with real-time patient data and enabling better care coordination. Finally, ongoing development efforts should prioritize a user-centric design approach, incorporating input and feedback from both patients and health care providers to maintain the app’s intuitiveness, accessibility, and alignment with evolving user needs.

### Conclusions

This usability study assessed the “MyPal for adults” app with a diverse user group, including individuals with chronic diseases, and patients with CLL. Participants generally had positive initial impressions of the app’s design and organization, though some preferred a more modern interface. Although certain modules posed usability challenges, the ePRO modules were well-received. However, enhancing learnability, personalization, and addressing technical issues in some tasks is essential. This study emphasizes the importance of iterative, user-centered development to improve the app’s usability and UX. Notably, older users favored simplicity, while younger users sought a contemporary design, indicating room for age-specific improvements. Given the app’s role in palliative cancer care, effective ePRO implementation and thoughtful translation of paper-based PROs into digital formats are critical. Ensuring timely health care provider responses to patient reports is vital. These findings offer valuable insights for refining the MyPal app and may inform other self-management mHealth systems in palliative cancer care.

## Supplementary material

10.2196/57342 Multimedia Appendix 1 Questions asked during the preparatory steps.

10.2196/57342 Multimedia Appendix 2 Usage scenario and task list.

## Abbreviations

CLL: chronic lymphocytic leukemia
ELLOK: Greek Federation of Cancer Patients
ePRO: electronic patient-reported outcome
HCP: health care professional
HM: hematological malignancy
ISO: International Organization for Standardization
mHealth: mobile health
PSSUQ: Post-Study System Usability Questionnaire
QoL: quality of life
SUS: System Usability Scale
UEQ: User Experience Questionnaire
UI: user interface
UX: user experience
