# Supplementary material for: Usability and User Experience Testing of a Co-Designed Electronic Patient-Reported Outcomes App (“MyPal for Adults”) for Palliative Cancer Care: Mixed Methods Study
Source: JMIR Hum Factors. 2025 Apr 24;12:e57342. doi: 10.2196/57342 (PMC12045520; doi:10.2196/57342)
Supplement: Multimedia Appendix 1 [file humanfactors-v12-e57342-s001.docx]

1. On a scale of 1(Poor) - 5 (Excellent) How would you rate your experience using applications?
2. What kind of things do you do regularly with your mobile phone?
3. Have you used an app to search for information about your health?
4. Tell me about the last time you used a health-related app. What was it and why?
5. Have you used an application similar to MyPal for adults in the past? (Yes, No, Not Sure)
