# Supplementary material for: Usability and User Experience Testing of a Co-Designed Electronic Patient-Reported Outcomes App (“MyPal for Adults”) for Palliative Cancer Care: Mixed Methods Study
Source: JMIR Hum Factors. 2025 Apr 24;12:e57342. doi: 10.2196/57342 (PMC12045520; doi:10.2196/57342)
Supplement: Multimedia Appendix 2 [file humanfactors-v12-e57342-s002.docx]

**Usage Scenario**: You are a patient who has been diagnosed with CLL for which you are receiving medication. However, there are no serious health implications for you at this moment, but you need to monitor it closely. Your doctor has informed you about the MyPal application and the smartwatch device that accompanies it. You already know that it is primarily a questionnaire application designed to help organize health information for both you and your doctor. It also includes some additional features. The questionnaires used in MyPal are standardized, meaning they have been designed by professionals for the specific purposes we use them for and are activated based on predetermined schedules. Specifically, there are weekly and monthly questionnaires that are sent to you to be completed within specific timeframes to ensure their timeliness. Other features include the spontaneous reporting of symptoms, medication reminders, the personalized MyPal search engine, and the display of your activity in steps and sleep quality on a chart.

And now with that information in mind let’s begin…

**Login and initial settings**

1. “You were given a username, a password and a MyPal password before our session. Have them available! Locate and open the application and tell us your opinion about the application icon.”
   - On a scale of 1(Very unsatisfied) - 7 (Very satisfied) where would you rate this task’s content in terms of experience/satisfaction?

**App Registration Process**

1. “You have started the registration process. Follow each step of the process and tell us what you think?”

**Custom personalization surveys and ePROs impression**

1. “You have reached a MyPal questionnaire. Enter your answer and let us know of your impressions?”
   - On a scale of 1(Very unsatisfied) - 7 (Very satisfied) where would you rate this task in terms of experience/satisfaction?
2. “Suppose you feel offended by a question and do not want to answer it. What would you do?”

**Smartwatch pairing procedure**

1. “You were also given a fitbit username and password. Use them to connect to the fitbit server.”
   - On a scale of 1(Very unsatisfied) - 7 (Very satisfied) where would you rate the app registration process in terms of experience/satisfaction?

**Home Screen impressions**

1. “Scroll up and down on this screen and let us know what you think of your first impression of the overall design?”
   - “What do you think is the purpose of this page?”
   - “Do you find this application reliable?”
     1. “If not, what would you expect to see from a trusted application?”
   - On a scale of 1(Very unsatisfied) - 7 (Very satisfied) where would you rate this task’s content in terms of experience/satisfaction?

**Questionnaire Module**

1. “You want to find and see your already answered questionnaires”
2. Now that you are in the questionnaire section, what are your impressions on this page? What can you do on this page?
   - On a scale of 1(Very unsatisfied) - 7 (Very satisfied) where would you rate this task’s content in terms of experience/satisfaction?

**Fitbit Module**

1. You want to find and view your fitbit data
2. Now that you are in the fitbit module, what are your impressions of this screen? What can you do here?
   - On a scale of 1(Very unsatisfied) - 7 (Very satisfied) where would you rate this task’s content in terms of experience/satisfaction?

**Drug Module**

1. You are about to start a new medication and your doctor advised you to register it in the MyPal application. Add your medication called ibuprofen with instructions for taking it: twice a day on a full stomach.
   - On a scale of 1(Very difficult) - 5 (Very easy) where would you rate this task in terms of difficulty?
   - On a scale of 1(Very unsatisfied) - 7 (Very satisfied) where would you rate this task’s content in terms of experience/satisfaction?
2. You tend to forget, so you'll want to set medication reminders.
   - On a scale of 1(Very difficult) - 5 (Very easy) where would you rate this task in terms of difficulty?
   - On a scale of 1(Very unsatisfied) - 7 (Very satisfied) where would you rate this task’s content in terms of experience/satisfaction?

**Symptom Report Module (1/2)**

1. You noticed a strange rash on your hand a few days after the medicine and you want to report it.
   - On a scale of 1(Very difficult) - 5 (Very easy) where would you rate this task in terms of difficulty?
   - On a scale of 1(Very unsatisfied) - 7 (Very satisfied) where would you rate this task’s content in terms of experience/satisfaction?

**Patient tailored disease-related information search engine**

1. You tend to worry a little about your rash. Your doctor has informed you that you can search for accurate information tailored to you through the MyPal search engine.
   - On a scale of 1(Very difficult) - 5 (Very easy) where would you rate this task in terms of difficulty?
   - On a scale of 1(Very unsatisfied) - 7 (Very satisfied) where would you rate this task’s content in terms of experience/satisfaction?

**Symptom Report Module (2/2)**

1. You want to make sure the information you entered about the symptom you mentioned earlier is accurate?
   - On a scale of 1(Very difficult) - 5 (Very easy) where would you rate this task in terms of difficulty?
   - On a scale of 1(Very unsatisfied) - 7 (Very satisfied) where would you rate this task’s content in terms of experience/satisfaction?

**Emergency Communication details**

1. You are curious to locate the contact details of the clinic
   - On a scale of 1(Very difficult) - 5 (Very easy) where would you rate this task in terms of difficulty
